# How to Manage the Suffering of the Patient and the Family in the Final Stage of Life: A Qualitative Study

**DOI:** 10.3390/nursrep13040141

**Published:** 2023-12-06

**Authors:** E. Begoña García-Navarro, Sonia Garcia Navarro, María José Cáceres-Titos

**Affiliations:** 1 Department of Nursing, University of Huelva, 21007 Huelva, Spain; sonia.garcia@denf.uhu.es (S.G.N.); mariajose.caceres@denf.uhu.es (M.J.C.-T.); 2 Research Group ESEIS, Research Center COIDESO, University of Huelva, 21007 Huelva, Spain; 3 Huelva Costa Health District, Junta de Andalucía, 21003 Huelva, Spain

**Keywords:** palliative care, end-of-life care, chronicity, aging, elderly care, nursing homes

## Abstract

Background: The end of life and death have changed from being issues managed within the family, assumed as part of life, to occur within health institutions for the majority of patients. The amount of patients dying at home has decreased, and the roles of families and communities in death and dying have become involuted, threatening related traditions and knowledge. As a result, a need to promote the end of life at home in this new self-serving society has arisen. In that context, the main objective of this study was to find out what patients and their families need during the end-of-life process in order to feel effectively accompanied at this time. Methods: With that objective, a descriptive qualitative study was conducted via the content analysis of data from semi-structured interviews and focus groups. This research adhered to the COREQ guidelines. The sample consisted of 36 informants selected via intentional sampling of family members and patients integrated into the Comprehensive Palliative Care Process (PAI Paliativos). Results: The results suggest the existence of several common needs such as communication and presence, including the conspiracy of silence as an important factor generating suffering for both. However, there are specific needs such as autonomy, dignity, and respect for patients, which must be taken into account. Conclusions: The results of this study will allow us to establish intervention strategies for effective accompaniment of patients’ family members at the end of life and the avoidance of ethnocentrism in this process. This study was retrospectively registered with the (nursrep-1194226) on the (21 April 2023).

## 1. Introduction

Healthcare for terminally ill patients and their families is an issue that is becoming increasingly important for the health system and society as a whole [1]. The progressive increase in the population and its life expectancy, together with scientific and technical advances, is generating an increase in morbidity and mortality due to cancer and other pathologies occurring in the last years of life [2]. The social reaction to this phenomenon is aggravated by the new family dynamics where the availability of informal caregivers is limited, attributable to changes in sociodemographic patterns (progressive decrease in relatives in the following generations and incorporation of women into working life, among others) [3].

Given that the institutional response to the problems relating to end-of-life care is insufficient, we are faced with the suffering process of the patient and the family, as well as the increasing demand for care that is not being met [4].

The end of life, i.e., death, has changed from being an issue that was managed within the family, assumed as part of life, to being an exclusive domain of the health system. These days, patients rarely die at home, and so the roles of families and communities have receded as death and dying have become unknown. Skills, traditions, and knowledge are lost along with an externalization of the end of life [5]. As the idea of death is a cultural product of society, the meaning of death affects associated fears, beliefs, hopes, and orientations. This construct is not instinctive, but is apprehended through public symbols, as well as a person’s own biography [6]. Thus, the prevailing system of death in society mediates the experience of dying. Since most human behavior is a product of cultural norms and values, social structures, and interaction with other members of the society in which one lives, dying and death are defined by the specific time and place in which they occur [7]. From a more individual point of view, the concept of death for the patient and their family is not only determined by cultural norms but is also influenced by the patient’s own biography and the dynamics of family accompaniment [8]. Several authors [9,10,11] have indicated in their research that the key to the paradigm shift in the phenomenon of death, as well as the suffering of the patient and their family, is the prevailing social trend for people to be more self-serving than in the past, according to which we prefer material possessions and achieving them with great immediacy over personal growth, together with having a great social intolerance to frustrating situations, along with an absence of education about death. All this is manifested in the individual sphere through suffering, and a high demand for care accompanies many people in their terminal stage [12].

During this final process, it is essential to focus care on the family unit, i.e., the patient and family, supporting caregivers during this stage. The approach to this care unit should not only offer resources to cope with understanding and alleviating physical symptoms but should also emphasize support for emotional management and mediation with the patient in order to avoid complicated grief [13]. On the other hand, we must not forget to support the families’ role as caregivers in the best possible way, helping them to make decisions and empowering their role.

Accompanying suffering is not an easy task and requires skills, abilities, and knowledge, as well as personal development. Identifying and dealing with suffering is one of the main functions of healthcare professionals in the development of their clinical practice. Being better able to identify what the patient and family need in this process would allow the healthcare professionals to accompany them based on the needs of the care unit and not in an ethnocentric way. With that aim, the main objective of this study was to find out what patients and families need in the end-of-life process in order to feel effectively accompanied.

## 2. Materials and Methods

The general objective of this research was to understand the needs of patients and their families during the end-of-life process in order to feel accompanied.

As specific objectives, it was proposed that we identify the needs of patients in the process of accompaniment in both the professional and family spheres, describe the needs of the family of patients who are in the final stage with respect to the patients themselves and healthcare professionals, and find out how the patient and family perceive professional accompaniment in this process.

### 2.1. Methodology

This study was carried out using a descriptive qualitative design of a phenomenological nature. This approach allowed us to explore and describe in depth the participants’ experiences and perceptions of the end-of-life process. For the development of this research, the guidelines of the COREQ were followed [14].

### 2.2. Participants

The sample was composed of 36 informants selected through purposive sampling in patients integrated in the Process of Integrated Palliative Care Attention (PAI Palliatives) of the Andalusian government, in the province of Huelva. A process of searching for patients and relatives of independent contacts was carried out in different areas of the province (rural and urban). This allowed us to maximize the capture of different experiences, as well as establish a more heterogeneous and more enriching discourse for the following analysis. The general inclusion criterion was that patients and relatives were informed of the process and integrated into the Expanded Programme on Palliative Care (PAI). The selection of the most illustrative profiles of informants to be interviewed was made by the nurses of the palliative care team of the reference hospital. The completion of the data collection process was determined following the principle of theoretical saturation [15]. Within our sample, 16 were terminally ill patients in advanced stages and 20 were direct relatives, who voluntarily agreed to participate in the study after reading the Patient Information Sheet and signing their informed consent.

This study followed the international ethical recommendations set out in the Declaration of Helsinki. All personal information provided was stored in a way that complied with the legal requirements for the protection of personal data and the guarantee of digital rights (Organic Law 15/1999 of 13 December 1999 and Organic Law 3/2018 of 5 December 2018). This project was approved by the Ethics Committee of the Andalusian Government (PEIBA).

In order to obtain a holistic understanding of the phenomenon, a methodological triad based on three complementary techniques was used: non-participant observation, in-depth interviews, and focus groups, carried out face-to-face in a neutral environment and recorded in audio and video.

The non-participant observation was carried out directly by one of the researchers, allowing us to capture aspects difficult to obtain through other techniques. The entire procedure described above was audio and video recorded by another member of the research team who also took field notes. The interviews, based on a thematic guide developed by the research team, lasted a maximum of 70 min and always began with the same main questions: Do you feel accompanied during this process? What would you need to be accompanied? The interviewee was never directed in their responses; the informants’ freedom of speech was guaranteed, just redirecting in some moments to continue with the content guide until the saturation of the discourse. During this interview, participants expressed their experiences in a free and detailed manner, as well as their perceptions about the needs and difficulties they faced during the end-of-life suffering process.

Subsequently, two focus groups were conducted with 10 participants each, following the same content guide and the same starting questions, with an average duration of 80 min. We developed this technique with the aim of encouraging interaction and discussion among the participants, allowing us to obtain information from the interaction of their discourses. Data collection continued until the theoretical saturation of the discourse was reached and until no new perspectives or relevant information on the phenomenon were obtained.

### 2.3. Analysis

To analyze the discourse obtained in the interviews, the model described by Taylor-Bodgan [16] was used in order to understand the experiences and perspectives of the interviewees. All focus group interviews and recordings were transcribed verbatim to maintain data fidelity. Participants were assigned an alphanumeric code consisting of “PA” in the case of patients and “AF” in the case of relatives, followed by a number assigned consecutively in order of interview.

Through deductive coding, the categories and emerging issues related to the phenomenon studied were identified and grouped. After the first general reading of the transcripts, in which several members of the team participated, a first identification of emerging categories was carried out. This first confrontation of categories served to reach a consensus on criteria for the coding process and thematic units of interest. Subsequently, another member of the research team collated the emerging categories developed. Repeated use of the same codes–dimensions by different members of the research team (blind analysis) indicated that analysis got to the essence and exposed the meaning of the studied phenomenon.

Methodological rigor was achieved by following requisites for verification, validation, and validity described by Meadows and Morse [17]. Verification was achieved during the planning and informant recruitment phase, which also included the delegation of tasks to different members of the research team. Validation was achieved through the different methods of data collection (interviews, focus group, and observation of field notes). Data analysis and coding were conducted by the most experienced researcher and cross-checked by another team member. Internal validity of the study was achieved by cross-referencing with a research team member who did not belong to the same research group but had expertise in the subject matter.

Aplasia software was used to facilitate the analysis of qualitative data. This program made it possible to organize and analyze the transcripts, notes, and categories that were identified. These provided the base unit of analysis, composed of several lines or sentences exposing a central idea extracted from the interviews. Preliminary categorizations were performed manually using ©2023 ATLAS.ti Scientific Software version 23 Development GmbH (Berlin, Germany).

The results of the analysis were carefully interpreted to identify patterns, relationships, and meanings of the experiences of the informants.

## 3. Results

The study population consisted of a total of 36 key informants. Of these, 24 informants were interviewed: 10 patients (PA1-PA10) and 14 family members (FA1-FA14). Additionally, 12 informants participated in two focus groups: one (FG1) for the patients (PA11-PA16) and the other (FG2) for the family members (FA15-FA20).

Regarding sociodemographic characteristics, the average age of the interviewed patients was 56.3 years old (SD = 6.93). Among the family members, the average age was 41.5 years old (SD = 7.46). In terms of gender, there were 11 female patients and 5 male patients, while among the family members, there were 11 females and 9 males. This distribution ensured an equal representation of both patient and family perspectives in the qualitative analysis of the study.

The findings presented in this study provide a thorough perspective on the perceived demands of both patients and their families during end-of-life distress. These needs are categorized into six dimensions relevant to the patient, also showing the lists of techniques used (see Table 1), and five to the family (see Table 2), covering various spheres: physical, emotional, spiritual, and social, while reflecting the complexity and sensitivity inherent in this critical phase. The emerging results obtained are highlighted in grey.

**Figure 1:**
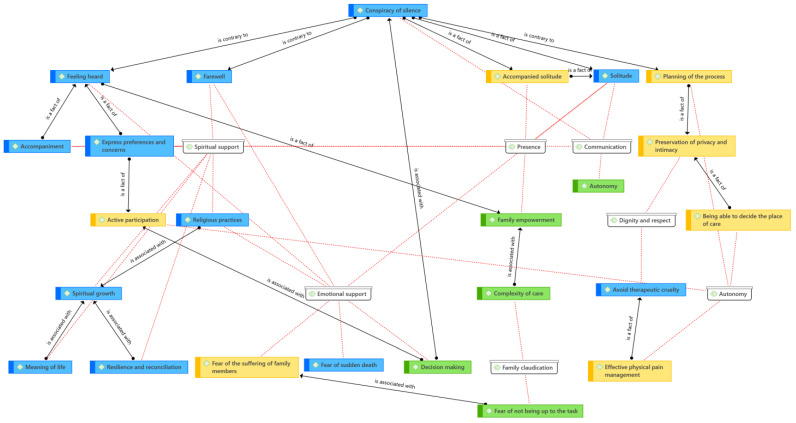
Relationships between dimensions and patient and family codes (Atlas ti. V.23). Source: own elaboration.

For both the patient and the family, common dimensions associated with the end-of-life suffering process and addressing their needs were identified. These dimensions are interrelated in Figure 1, with the dimensions shown in white, the common codes in blue, the patient-specific ones in yellow, and the family-specific ones in green (see Figure 1).

The first dimension was presence. Both patients and family members highlighted the importance of accompaniment at this stage, reinforcing the idea that social and emotional support is essential to mitigate the loneliness and isolation often experienced in this process. However, while this dimension was common for both patients and relatives, nuances emerged in the approach to this process. Several patients expressed the experience of “accompanied solitude”, which suggests that it is not only important to be accompanied but also to feel truly accompanied, beyond a mere physical presence (see Table 3).

On the other hand, family members emphasized the need to find resources and tools that provide comfort, strength, and support in this situation. This indicates that, although they are accompanying the patient, they also face emotional challenges and need support to cope with this process of suffering.

Communication proved to be a common need for both patients and their families. However, this need is frequently interrupted by what is known as the “conspiracy of silence”. While patients expressed a desire to be able to express their preferences and concerns without restrictions, it is challenging for the family to talk about death. In this sense, the need to overcome the taboo and fears associated with the dying process is presented, and the need for open communication between the patient, family, and healthcare workers (see Table 4).

The dimension of spiritual support becomes an essential element for many patients who are approaching the end of life, allowing a space for reflection and search for sense and meaning in their lives. Feeling accompanied on their journey, both by health professionals and by family and friends, can provide them with a sense of connection, comfort, refuge, and emotional support during this period. Resilience and reconciliation also emerge as important factors in the dimension of spiritual support. Patients may face complex emotions as they prepare for the end of their lives, and the ability to adapt and find strength in the midst of these circumstances can be crucial to their well-being. In addition, spiritual growth becomes a meaningful pursuit for many patients. This spiritual growth can manifest itself in a variety of ways, such as a greater understanding of life and death, a strengthening of religious beliefs and practices, or a deeper connection with the world and others.

As for family members, they also seek to find meaning and meaning in the process of caring for a loved one at this stage. Spiritual support becomes a tool to face the difficulties and challenges involved in caring for a patient at the end of life. Spiritual accompaniment is also important for family members, as they may experience a deep need to find comfort and strength during this stage. Resilience and reconciliation are fundamental aspects to facing the emotions and tensions associated with the care process and the proximity of death. Finally, the possibility of saying goodbye becomes a significant element in the spiritual dimension for both patients and their families. The act of saying goodbye can be a time of connection and emotional healing for both parties, allowing them to express their feelings and say goodbye with love and respect (see Table 5).

The emotional support dimension reflects the concerns and fears experienced by patients and family members. In the case of patients, this dimension reflects two of the most frequent concerns: the fear and uncertainty associated with the moment of death and, above all, the suffering of their loved ones. The desire to feel heard is another crucial need identified in this dimension. Patients may experience a range of emotions and thoughts that they wish to express to those around them, whether to their family members or healthcare professionals. Feeling heard and understood in this process can bring them comfort and emotional validation, which contributes to their emotional well-being. In addition, avoiding the feeling of loneliness is another important need in this dimension of emotional support. As patients face the end of their life, they may experience an increased sense of isolation and emotional disconnection. Emotional support and empathic accompaniment can help alleviate this feeling of loneliness and give them a sense of connection and closeness with their loved ones. In this context, religious practices and the possibility of saying goodbye become important sources of emotional support for patients. Religious practices can provide comfort and a sense of connection to the spiritual world, which can be especially meaningful for those who find comfort in their faith during this stage. Likewise, the ability to say goodbye to loved ones gives patients emotional closure and the opportunity to express their love and affection before leaving, which can be comforting and meaningful to them.

On the other hand, family members also expressed fears and concerns related to the patient’s care process. The fear of the sudden death of a loved one and the desire to feel heard and understood in their concerns and needs are the key aspects for family members. In addition, the ability to participate in decision-making related to patient care gives them a greater sense of control and empowerment in this challenging situation (see Table 6).

Once the common dimensions were analyzed, we found two differences concerning the needs of patients. The autonomy dimension underscores the importance of enabling patients to make decisions about their care and plan the final process. Effective physical pain management, the ability to decide where to receive care, and actively participating in their care are essential elements to respect their autonomy and dignity at this important stage. Finally, the dimension of dignity and respect reveals how patients seek to preserve their privacy and intimacy and avoid therapeutic cruelty, which shows the need to receive respectful and personalized care.

PA4: “*The idea of having control over my own attention gives me a sense of tranquility and security. I want to be involved in the planning process and make informed decisions about my care*”.

Regarding the family, we highlight a specific dimension, family incapacity, which shows how family members face difficulties in caring for patients at this stage, such as fear of not being up to the demands of care and complexity involved in providing the necessary care.

FA9: “*Thinking every moment you can you have stopped doing something he needed, that some of your words could hurt him more than help him… not knowing what he was thinking when his gaze was lost and he was with you physically but not mentally… It gives a lot of vertigo to think that you do not do it well. You are very afraid*.

FA11: “*Having to put medication when he is restless, when you see that he cannot be in any way, knowing or thinking that if you give the medication you can slow him to death…*”.

FA19: “*Take care of the tracheostomy, the PEG, give the medication, help the nurse to heal the wounds… I was not prepared for all this but in the end you do it with the help of professionals because they make the difficult easy for you…”. If I look back and think about it……… I don’t know how I could do it, my house looked like an ICU with so much apparatus*.

Taken together, these results show that the process of suffering at the end of life is complex and affects both patients and their families. The needs identified in both patients and family members are configured as the central axis to provide a dignified final process, based on holistic and competent care by health professionals. These findings have significant implications for improving healthcare services and the training of health professionals, ensuring a more humane and sensitive approach for those facing the end of their life and of that of their loved ones. In addition, the findings highlight the need to provide support to both patients and their families, recognizing the importance of emotional and spiritual well-being in this transition process.

## 4. Discussion

This study provides deeper insight into the perceived needs of the patient and family during the end-of-life suffering process. The results highlight the importance of person-centered care, taking into account the family as an integral part of the patient’s care. In addition, it is really important to have a comprehensive approach to the care during the end-of-life process, with a compassionate vision of professionals as an integral and necessary part of the emotional and spiritual accompaniment of the patient–family dyad. In this sense, we agree with the description that the WHO [1] gives for the approach to suffering, since it involves dealing with problems that are not limited to physical symptoms but also extend to the emotional, psychosocial, and spiritual sphere, as our informants referred to in both populations. The needs of the patients and their families identified in this study cover all dimensions that may be addressed by health professionals. Both populations described the need to ensure a spiritual approach from all actors in the process. These results converge with the conclusions of research by other leading authors in this area [18,19] that refers to the need to provide comprehensive care at the end of life to alleviate suffering (physical, emotional, social, and spiritual) and dignify the dying process.

The results of this research evoke the need expressed by both patients and relatives for professional presence as a therapeutic tool during the suffering process. Along the same lines, Benito [20] identified presence as a powerful therapeutic tool for the healthcare professional who accompanies people during suffering. This optimizes the therapeutic relationship, facilitating the experience between the clinician and the person accompanied. Benito describes presence as “being and meeting the person who suffers with “*your whole being”* and being present during the intervention at all levels, reaching the physical, emotional, cognitive, relational and spiritual spheres”. On the other hand, the nursing profession recognizes presence as its own intervention [6,7] and defines it as staying with other people during times of need, both physically and psychologically, and identifies activities such as staying with the patient to transmit a feeling of security and confidence, showing an attitude of acceptance, listening to the patient’s concerns, or being physically available to help. Some studies reveal the helping relationship as a nursing practice by the nature of the profession [21], understanding nursing as the integral care of the person and the helping relationship as the essence of the accompaniment of people who suffer.

The informants of our study corroborate the felt need for nursing presence, with both populations describing it as an element favoring effective accompaniment. Presence is described in our study as a protective factor for the loneliness felt by both the patient and the family and as a driving force in coping with the process. The results of this research speak of suffering in both the patient and family populations. Cecily Saunders [22] in the 1970s defined the concept of “total pain” to describe the layers of physical, emotional, social, and spiritual (existential) suffering that accompany the diagnosis of advanced cancer. This concept is understood by other authors as not limited only to the palliative process [23] and is described in the proximity of death as a suffering for the sick person in different areas, identifying the process of dying as an experience of illness, a suffering, and a social dysfunction that involves different forms of the concept of suffering [24] and that must be addressed in a comprehensive manner.

This research highlights the need to accompany the suffering of patients and families in the final process, as both populations describe in their discourse a need for a comprehensive approach taking into account all the levels of expression of suffering previously referred to by different authors [24,25], emphasizing social dysfunction during the final process and the experience of illness. Presence and communication, or lack thereof, are intimately linked as part of the helping relationship during the accompaniment of people who are suffering. In the results of this study, the expressions of the informants of both populations describe how communication can be considered an essential part of the human being and needs to be satisfied by the professionals who accompany them during the end-of-life process, both in the patient and the family.

Communication is the fundamental tool of healthcare professionals, and this tool becomes much more powerful when we are facing communication with the patient and family during the end-of-life process. The way to communicate with a palliative patient and their family will define the type of relationship that is established. This situation requires that the health professionals who attend them have skills for physical, emotional, and spiritual accompaniment. Communication acquires at this time the essence of integral care and is part of the established means of the helping relationship. In our study, the need for professional communication is evident as a tool to help expression, both by people during the end-of-life process and by the family members who care for them. This communication allows caregivers to feel safe in care while helping them to face their fears in the face of the death of the loved one and allows them to grow during the process of acceptance of it [25,26]. The patient’s family needs to feel heard and validated by health professionals. This need was reflected in our study by both family members and patients, with the latter concerned that the feelings of their loved ones were welcomed and accompanied.

The conspiracy of silence is often part of the accompaniment process. The pact of silence is a communication barrier with the patient caused by an implicit or explicit agreement that the family reaches with the health professional to omit information provided to the patient in order to protect them from the impact that knowing about the status of their condition may have on their life [27]. Patients who are in the final process of their life have many fears and uncertainties regarding their process and prognosis, so it is necessary to express their emotions and feelings with their loved ones and the professionals who accompany them. However, the pact of silence in these circumstances causes an increase in their perceived loneliness and social isolation as a result of them not feeling supported by the people who accompany them. This situation increases the patient’s suffering, worsening their quality of life and making it difficult for them to cope and accept the process [28,29]. This study verifies the need felt by patients for a veracity of the information provided by the people who accompany them, both by their relatives and by professionals. Patients associate the veracity of the information and the ability to access it with an improvement in their autonomy in decision-making and think of this as a protective factor in their acceptance of the process. The information must be adapted to the patient and their end-of-life stage. Chochinov [30] affirms the need for constant communication of professionals with the patient regarding their values and beliefs, particularly given the potential changes in the latter during the process of their disease, and making decisions based on symptoms and prognostic beliefs. On the other hand, the relatives of the patients understand there is a need to strengthen this pact of silence for different reasons, some related to the protection from added suffering by avoiding expressing the closeness of death with their own family and on other occasions as a protective factor for the patient, as was reflected in the present study and in other research [27,28,30]. Finally, some caregivers interviewed identified throughout the professional accompaniment the difficulties offered by this lack of communication with their family member.

Family members of patients who are in an end-of-life process perceive the need to feel accompanied in the same way as the patient, as they adopt the pain of the person they care for as their own, along with their beliefs and ideologies on how to find meaning in care. In this sense, we agree with Victor Frankl in terms of the idea of the fundamental search of the human being [12]. Frankl in his work suggests that this search is not directed to material goods, power, or prestige, but instead to an experience of meaning, affirming that the ultimate freedom of the human being is to choose their attitude to any circumstance, which cannot be taken away by anyone. The professionals who accompany patients and their families during the process of dying can help them find that attitude to difficult circumstances (meaning of life) through those things created, achieved, and loved by the person and that for them acquire a meaning as a legacy. This research presents in its testimonies this search in both patients and relatives finding the desired well-being.

It is important to conclude this discussion with an idea that was already outlined at the beginning, the need expressed in both patients and relatives for a spiritual approach during the final stage of life. This idea has generated great interest in recent years since it is not considered part of the competence of health professionals to address this need. However, there are different studies that advocate for addressing the spiritual need [31,32,33,34,35,36] in the patient during the end-of-life process, proposing that this should be included in training for healthcare professionals. The proximity of death is one of the moments of spiritual awakening of the person and we must guarantee an associated attention to the people we accompany. Our research coincides with other studies that identify the need expressed by patients to “be cared for by professionals capable of humanely alleviating the physical, emotional, and spiritual pain they experience during the dying process” [36].

As a limitation, we must highlight that having held internal presumptions about the phenomenon of study due to the expertise of the authors in this subject, despite having made use of reflexivity, has limited some of the stages of the validation process. Furthermore, due to the terminal nature of the patients included in this study, it was very difficult to access the informants to obtain feedback from the data analysis.

## 5. Conclusions

End-of-life care represents a major challenge in our society due to the low percentage (14%) of patients with palliative needs who that are met. This need for palliative care will grow due to the increase in noncommunicable diseases and the aging of the world population. Palliative care by specialist professionals is a component of the provision of palliative care services. However, a sustainable, quality, and accessible palliative care system must be integrated into the context of primary healthcare, community, and home care, and supportive care providers, such as family members and community volunteers. The provision of palliative care services should be considered an ethical duty of health professionals.

Taking into account this context, it is necessary to establish measures that guarantee palliative care at all levels of care responsive to all chronic conditions. The professionals who accompany patients and family members in the end-of-life process must have obtained specific competencies regarding communication, presence, compassion, and spirituality, necessary for a comprehensive approach to this process and prevention of ethnocentrism at the end of life.

We believe that this study provides findings helpful to identify the competencies necessary for health professionals to comprehensively address patients and families in need of palliative care, ensuring a more humanized approach at all levels of care.

## Figures and Tables

**Table 1:** Needs detected by the patients themselves in the end-of-life process according to different techniques.

Dimension	Codes	Dating	Non-Participant Observation	Interview	Discussion Group
Presence	Accompaniment	12	Yes	Yes	Yes
	Accompanied solitude	8	Yes	Yes	
Communication	Conspiracy of silence	10		Yes	Yes
	Express preferences and concerns	14		Yes	Yes
	Solitude	6	Yes	Yes	Yes
Spiritual support	Meaning of life	10		Yes	Yes
	Accompaniment	11	Yes	Yes	Yes
	Resilience and reconciliation	7		Yes	Yes
	Spiritual growth	6		Yes	
	Religious practices	13	Yes		
	Farewell	11		Yes	Yes
Emotional support	Fear of sudden death	8		Yes	
	Fear of the suffering of family members	5		Yes	Yes
	Feeling heard	12		Yes	
	Solitude	9	Yes	Yes	Yes
	Religious practices	7	Yes		
	Farewell	11		Yes	Yes
Autonomy	Effective physical pain management	6		Yes	
	Being able to decide the place of care	7		Yes	
	Active participation	9	Yes	Yes	
	Planning of the process (preparation of will and final wishes)	10			Yes
Dignity and respect	Preservation of privacy and intimacy	13	Yes	Yes	Yes
	Avoiding therapeutic cruelty	9		Yes	Yes

Source: own elaboration.

**Table 2:** Needs detected by relatives of patients in the end-of-life process according to different techniques.

Dimension	Codes	Dating	Non-Participant Observation	Interview	Discussion Group
Presence	Accompaniment	10	Yes	Yes	Yes
	Solitude	8	Yes	Yes	Yes
	Family empowerment	5		Yes	Yes
Communication	Conspiracy of silence	7		Yes	Yes
	Autonomy	6	Yes	Yes	Yes
Spiritual support	Sense of care/meaning of life	9		Yes	Yes
	Accompaniment	11	Yes	Yes	Yes
	Resilience and reconciliation	6		Yes	Yes
	Spiritual growth	5		Yes	
	Farewell	10		Yes	Yes
Emotional support	Fear of sudden death	7		Yes	Yes
	Feeling heard	9		Yes	
	Solitude	8	Yes	Yes	Yes
	Decision-making	7		Yes	
Family claudication	Fear of not being up to the task	4			Yes
	Complexity of care	5		Yes	

Source: own elaboration.

**Table 3:** Codes and discourse of patients and relatives on the need to be present during the end-of-life process.

Dimension	Codes	Dating	Informants	Discourse
Presence	Accompaniment	12	Patient	PA15: *“My family and friends are with me as much as they can, although sometimes I feel they take care of me with excessive caution and fear. I want to be seen as the person I always was, not just someone who is dying”.*
		10	Relative	FA1: *“When I’ve had any doubts, I have a phone to call, that… It’s very important. I remember the day the unbearable pain began, nothing made him calm it, knowing that he had them on the other end of the phone and that sweetness and security with which they talk to you… That’s priceless and allowed me to keep her at home, just the way she wanted”.* FA5: *“When they would come home and say, ‘You’re the best patient we have, you’re doing great… It gave him the energy he lacked to continue until the next visit”.*
	Solitude	8	Patient	PA15: *“As the disease progressed, the feeling of isolation grew stronger. At times, it was as if I was trapped in a world apart, with questions and fears that seemed to have no answer. However, the constant presence of my family and the unconditional support of the medical team brought me invaluable comfort. Knowing that they were by my side, sharing every step of this path, made the burden more bearable. I didn’t feel alone in this fight, and that made all the difference”.*
		8	Relative	FA7: *“When everything got worse they called me almost every day and they came very often both they and the professionals of the health center,* *It helped me to feel accompanied in this story in which you have so many doubts that you never know if you are doing everything right. They have helped me a lot and although it has been difficult, it is more bearable not to feel alone”*
	Family empowerment	5	Relative	FA18: *“Thanks to my care I had her for seven months”, “when I came to the caregiver workshop at the health center, I realized that I was doing very well”, “I healed her injuries… as you have taught me”, “I slept with her every day and hugged her so she did not feel alone”, “I wanted her to leave with the greatest dignity in the world: I put her candles that smelled, the soft music… so that she would perceive peace and tranquility at home”, “I washed her, I dressed her…”.* FA15: *“From the first day you came to my house, everything started to change, the whole family started to change, my children were a little separated from their father’s illness, they didn’t expect it to be so advanced, when he said he was worried that I would be left alone… That caused an awakening in my children and although the end has been the same… The journey has been very different, they have not left their father or me alone. I can only thank you”.* FA6: *“There will come a time when she will not be able to move from the bed, the nurse told me one of the first days: I fell badly, I thought it was an exaggerated but over time I understood that this was the case and that at this time and as the nurse told me what I had left was to help my mother-in-law be happy and so it was between all of us we got it to be happy during the 11 months it lasted, we spent a Christmas eating the grapes in the room with her, we celebrated her birthday…”. “You helped us to live with her and enjoy those moments. She was happy and we were happy with her”.*

Source: own elaboration.

**Table 4:** Codes and discourse of patients and relatives on the need for communication during the end-of-life process.

Dimension	Codes	Cites	Informant	Discourse
Communication	Conspiracy of silence	10	Patient	PA14: “*From the beginning, I felt like something didn’t quite fit. He didn’t have much information. There have been times when he has felt that things were being saved. I understand that they wanted to protect me, but the lack of transparency also led to anxiety and confusion. I would have preferred to be spoken to sincerely to process reality together. In the end, open and honest communication is what is really needed to deal with this situation in the best possible way”.*
		7	Relative	FA3: *“I didn’t want her to find out anything so she wouldn’t suffer… what I found difficult to understand that she also knew everything without telling her… But everything comes when you have professionals next to you who know what they do. A disease like this does not let you see reality It is a very big suffering to see the person you love most in the world suffer, always keeping in mind that the end is near…”.* FA20*: “This lack of communication did not allow him or us to express ourselves, which made everything that was happening even more difficult…”.*
	Express preferences and concerns	14	Patient	PA3: *“I know that talking about death is difficult, but I want to express my thoughts and desires without restrictions, although I notice that sometimes they are not prepared to hear it”.*
	Loneliness	6	Patient	PA5: *“There were times when I craved a candid conversation about what was happening, but silence seemed to envelop everything. I understand everyone was trying to protect me”.*
	Autonomy	6	Family	FA13: *“That morning when the situation was no longer enough, they explained to my mother-in-law what was happening, the symptoms could not be controlled and the best option to reduce suffering was sedation. I shook my mother-in-law’s hand and asked her if she agreed. She nodded to me yes and it all started…. I didn’t want to (sob), we are very selfish and we want to keep her by our side despite everything but when you see her suffer so much… you understand that this cannot go on like this”.*

Source: own elaboration.

**Table 5:** Codes and discourse of patients and families on the need for spiritual support in the end-of-life process.

Dimension	Codes	Cites	Informant	Discourse
Spiritual support	Meaning of life	10	Patient	PA9: “*At times like this, you learn to appreciate each day, to value the little things. The only thing you care about is whether you’ve been happy and whether your family is okay… leaving that resolved, it doesn’t matter what”.*
		9	Relative	FA2: *“It helps us understand that life has a beginning and an end and how important it is to help close doors before we die…”.*
	Accompaniment	11	Patient	PA15: *“Being able to count on the support of the hospital chaplain, who was constantly by my side, listening to my concerns and reflections has given me comfort and helped me find peace in the midst of uncertainty”.*
		11	Relative	FA14: “*There will be no days in life to be thankful for that nurse’s words. It was like opening the window and getting a shot of fresh air with the smell of eucalyptus that opens your lungs”.*
	Resilience and reconciliation	7	Patient	PA11: *“Over time, difficult conversations became opportunities to heal and find understanding. Solving problems and talking to people I was angry with not only brought clarity and peace to my own heart, but also strengthened the bonds with those around me. It was a liberation to know that I left nothing unsaid”.*
		6	Relative	FA8: “*I was born into a religious family and I was the black sheep, I got into drugs… My father and my family never gave me up…. I fell again and a few days before I died and thanks to the intervention of the professionals who helped me understand the importance of saying goodbye to him and asking for forgiveness…”.*
	Spiritual growth	6	Patient	PA7: *“As I go through this stage of my life, I feel a deeper connection to my surroundings. I have learned to appreciate the small moments and to find comfort in nature and in the love of my family. I feel at peace and in harmony with everything around me”.*
		5	Relative	FA14: *“It helps us understand that life has a beginning and an end and how important it is to help close doors before we die…”.*
	Religious practices	13	Patient	PA16: “*Facing this difficult time has given me the opportunity to grow and understand more about life and death, and has brought me closer to my religious beliefs and practices”.*
	Farewell	11	Patient	PA2: *“Before I die, I would like to talk to my children, even the one we haven’t spoken to for a long time”.*
		10	Relative	FA6: “*I was able to say goodbye to him… and that makes me feel better, I did for him everything he wanted and how he wanted it”. But I was able to do all this because they helped me do it, they taught me all the time how to act, thanks to their advice, their calls, their hugs…* FA5: “*When I went to see him at the hospital he thanked me for being with him. This will be the last time, I wait for you even if you are not in a hurry. We hugged”*

Source: own elaboration.

**Table 6:** Codes and discourse of patients and relatives on the need for emotional support in the end-of-life process.

Dimension	Codes	Cites	Informant	Discourse
Emotional support	Fear of sudden death	8	Patient	PA9: *“I knew the situation was delicate, and at any moment things could take an unexpected turn. That uncertainty was terrifying. Not being able to prepare emotionally for what might happen generated an overwhelming sense of helplessness. We learned to live with fear, but also to find beauty in the midst of uncertainty”.*
		7	Relative	FA11: *“Lately I don’t go out on the street anymore, my neighbors bring me groceries, I don’t want to leave the house or leave it with my daughter. Thinking that when I come back I won’t be there prevents me from leaving the house”.*
	Fear of the suffering of family members	5	Patient	PA12: *“The fear of seeing my loved ones suffer because of me was a constant shadow in my heart”.*
	Feeling heard	12	Patient	PA4: *“There were so many thoughts, emotions and reflections that I needed to express. Feeling understood and validated became a deep need. I thank my medical team and my loved ones for giving me that space to share my concerns and wishes”.*
		9	Relative	*FA8: “It is very important that someone listens to you when you have so many fears and so many doubts. I have felt very accompanied”*
	Loneliness	9	Patient	PA8: “*I appreciate the emotional support I have received from my family and friends. Knowing that they are by my side gives me a sense of closeness and helps me cope with this sense of isolation”.*
		8	Relative	FA15: *“The toilets have worried a lot about me, they called me every week to see how everything was going and they always asked me how I was, that comforted me very much… to feel that someone cares about you in these moments when you feel so alone… it is very comforting”.*
	Religious practices	7	Patient	PA13: *“Praying every night and praying for my family is what comforts me the most. Knowing that I can die and that everything is going to be okay, that I will be reunited with my parents”.*
	Farewell	11	Patient	PA1: *“I felt the need to leave a legacy of love and caring, to make sure they knew how important they were to me. Although the sadness was palpable, there was also a sense of peace knowing that we were sharing these moments together, celebrating life”.*
	Decision-making	7	Relative	FA10: *“I made the decision and prepared the environment as she liked all her saints in the hairdresser, smell of incense and background her music. I felt very satisfied when it was all over because it had been done the way she liked it”.*

Source: own elaboration.

## Data Availability

Third-Party Data. Restrictions apply to the availability of these data. Data was obtained from anonymous informants and are available through the authors with the permission of the informants.

## Public Involvement Statement:

Patients and relatives participated in the research as the study population. They checked the understanding of the modified elements and the comments from a non-specialist perspective (GRIPP2).

## Guidelines and Standards Statement

This manuscript was drafted against the Consolidated criteria for reporting qualitative research (COREQ) for Qualitative research.
